# Interstitial Lung Disease in Firefighters: An Emerging Occupational Hazard

**DOI:** 10.3389/fmed.2022.864658

**Published:** 2022-03-21

**Authors:** Cathryn T. Lee, Iazsmin Bauer Ventura, E. Kate Phillips, Amy Leahy, Renea Jablonski, Steven Montner, Jonathan H. Chung, Rekha Vij, Ayodeji Adegunsoye, Mary E. Strek

**Affiliations:** ^1^Section of Pulmonary and Critical Care Medicine, Department of Medicine, University of Chicago, Chicago, IL, United States; ^2^Section of Rheumatology, Department of Medicine, University of Chicago, Chicago, IL, United States; ^3^Division of Pulmonary and Critical Care Medicine, Department of Medicine, Brigham and Women's Hospital, Boston, MA, United States; ^4^Section of Thoracic Radiology, Department of Radiology, University of Chicago, Chicago, IL, United States

**Keywords:** interstitial lung disease, occupational exposure, firefighting, interstitial lung disease risk, case series

## Abstract

**Introduction:**

Occupational risk factors for interstitial lung disease (ILD) are a remediable aspect of this progressive pulmonary disorder. The association between firefighting and ILD is unknown. Our objective was to assess the characteristics of firefighters with ILD from a large single-center ILD registry.

**Methods:**

The University of Chicago ILD database was reviewed for patients with a history of firefighting. Clinical information was abstracted from the medical record. The prevalence rate ratio of firefighters in the database compared to the baseline prevalence of firefighting in the Chicago metropolitan area was calculated via the Poisson distribution.

**Results:**

Nineteen firefighters were identified; all were men. A variety of ILD subtypes were seen across the cohort, including four patients with a diagnosis of connective tissue disease. Patients had mild forced vital capacity (FVC) and moderate diffusing capacity for carbon monoxide (DLCO) decrements on presentation; three patients died and two received lung transplantation over an average follow-up time of 76 months. Firefighters were seen at a greater proportion in the ILD registry than in the general population with a prevalence rate ratio of 3.98.

**Conclusions:**

Firefighting was overrepresented in our cohort compared to the general population, suggesting that there may be a causative association between firefighting and the presence of ILD. The wide variety of ILD subtypes observed suggest that all ILD patients should be asked about their occupational history. Further investigation to identify occupational exposures and determine the benefit of remediation is needed.

## Introduction

Interstitial lung disease (ILD) is a highly morbid, progressive, and often fatal pulmonary disorder with multiple potential etiologies. These can include autoimmune disease, inhalational exposures, and medications, although some patients ultimately have no cause found (1, 2). As treatments for ILD often slow, but do not stop, disease progression, efforts targeted toward the more proximate cause of ILD could result in improved clinical outcomes (3, 4). Thus, identifying and mitigating occupational inhalational exposures could represent a method of disease prevention and amelioration.

Firefighters' work exposure to toxic substances has resulted in an increased rate of chronic pulmonary and cardiovascular disease. Recently, first responders in the World Trade Center (WTC) attacks have been demonstrated to have a high rate of obstructive lung disease, autoimmune disease, sarcoidosis, and recent self-reports of pulmonary fibrosis, a devastating form of ILD (5–7). Little is known, however, about non-WTC firefighting and its association with subtypes of ILD formally diagnosed by a multidisciplinary team review and the possibility of concomitant autoimmune disease.

In this study, we reviewed a tertiary care center ILD database for subjects with an occupational history of firefighting. We hypothesized that firefighters would have a disproportionately high prevalence rate in our registry and that firefighters would exhibit a wide range of ILD subtypes, including those related to autoimmune disease. This would represent an important exposure association with ILD, with the potential to be a remediable cause of this often inexorably progressive disease.

## Methods

The University of Chicago ILD Natural History Database, a prospective registry approved by the University's Institutional Review Board (IRB #14163-A), was reviewed for patients with a history of firefighting. The electronic medical record of all patients enrolled in the registry from January 2007 to October 2019 was searched for a history of firefighting. Demographic and historical information, occupational histories, serologies, pulmonary function testing, and imaging reports were reviewed. Antinuclear antibody (ANA) was recorded as clinically significant if the titer was ≥1:320. Multidisciplinary ILD diagnosis (MDD) was conducted according to current American Thoracic Society/European Respiratory Society guidelines (8). Survival and lung transplantation status were ascertained and follow-up data was censored on November 1, 2019 or when a participant was lost to follow-up. Discrete variables are displayed as counts and percentages, while continuous variables are displayed as means with standard deviations.

Additionally, ascertainment of the number of employed firefighters in the Chicago-Naperville-Elgin, IL-IN-WI metropolitan area was obtained from the United States Bureau of Labor Statistics. The exact prevalence ratio was calculated comparing the prevalence of firefighting observed in ILD clinic versus the prevalence of firefighting in the general working population, and the probability of observing this prevalence ratio was calculated vs. the Poisson distribution of an observed variable compared to the expectation. Statistical analyses were conducted using Stata (9).

## Results

Nineteen firefighters were identified; all were men (Table 1). The mean age at time of presentation to clinic was 63 ± 11 years. Sixteen (84%) self-identified as white, while three (16%) self-identified as Black. Thirteen (68%) had a history of tobacco use with a median of 15 pack-years. Five (26%) reported a history of significant secondhand smoke exposure. None had a family history of ILD, while four (21%) had a family history of connective tissue disease (CTD). Comorbid conditions, including gastroesophageal reflux disease, were common.

**Table 1:** Cohort characteristics.

**Demographics**		**Exposure**		**Disease**
Age, mean ± SD	63 (11)	Ever Smoker, *n* (%)	13 (68%)	Autoantibodies, *n* (%)	10 (53%)
Male, *n* (%)	19 (100%)	Pack years, median (IQR)	15 (0–40)	ANA>1:320	6 (32%)
Race, *n* (%)		Secondhand smoke, *n* (%)	5 (26%)	SSA	2 (11%)
White	16 (84%)	Volunteer firefighter, *n* (%)	3 (16%)	Other	5 (26%)
Black	3 (16%)	Years worked, median (IQR)	28 (20–39)	ILD diagnosis, *n* (%)	
Asian	0 (0%)	Hospitalized after fire, *n* (%)	5 (26%)	IPF	10 (53%)
Family history of ILD, *n* (%)	0 (0%)	Still firefighting, *n* (%)	3 (16%)	CTD-ILD	4 (21%)
Family history of CTD, *n* (%)	4 (21%)	Other work exposures, *n* (%)	8 (42%)	CPFE	3 (17%)
CTD diagnosis, *n* (%)		Construction	4 (21%)	Unclassifiable	1 (5%)
MCTD	1 (5%)	Metal Work	2 (11%)	Sarcoidosis	1 (5%)
Autoimmune myositis	2 (11%)	Other	3 (16%)	Pathology, *n* (%)	
ANCA vasculitis	1 (5%)	Hobby exposures, *n* (%)	6 (32%)	UIP	2 (11%)
Comorbidities, *n* (%)		Bird or mold exposures, *n* (%)	7 (37%)	HP	1 (5%)
CAD	10 (53%)			UIP+OP	2 (11%)
COPD	4 (21%)			FVC %pred	72 (18)
Diabetes Mellitus	2 (11%)			DLCO	51 (16)
GERD	14 (74%)			Death	3 (16%)
				Lung Transplantation	2 (11%)

*ILD, interstitial lung disease; CTD, connective tissue disease; MCTD, mixed connective tissue disease; ANCA, Antineutrophil Cytoplasmic Antibodies; CAD, coronary artery disease; COPD, chronic obstructive pulmonary disease; GERD, gastroesophageal reflux disease; ANA, antinuclear antibodies; SSA, Sjogren's syndrome related antigen A autoantibodies; IPF, idiopathic pulmonary fibrosis; CPFE, combined pulmonary fibrosis and emphysema; UIP, usual interstitial pneumonia; HP, hypersensitivity pneumonitis; OP, organizing pneumonia; FVC, forced vital capacity; DLCO, diffusing capacity for carbon monoxide*.

Most had a history of longstanding firefighting. The median number of years working as a firefighter was 28, and five (26%) patients reported being hospitalized after exposure to a fire. Three (16%) patients were still firefighting at the time of clinical presentation. Co-exposures (not including tobacco) were common, with eight (42%) patients reporting an additional occupation that has been associated with ILD, six (36%) reporting a hobby that has been associated with ILD, and seven (37%) reporting other domestic mold or bird exposures.

A wide variety of ILD subtypes were observed among this cohort. Ten (53%) firefighters had idiopathic pulmonary fibrosis. Four (21%) patients with previously diagnosed CTD had CTD-ILD; two of those patients had a family history of CTD. Over half (53%) of patients had a positive autoantibody; one third of the cohort had an ANA titer greater than 1:320. Three were given a diagnosis of combined pulmonary fibrosis and emphysema (CPFE); all three of these patients had a history of smoking. One patient each had unclassifiable ILD and sarcoidosis. Five patients had surgical lung biopsy performed; of those, two (40%) had usual interstitial pneumonia (UIP) alone, two (40%) had UIP with organizing pneumonia, and one (20%) had hypersensitivity pneumonitis.

The mean forced vital capacity (FVC) at time of clinical presentation was 72% predicted, while the mean diffusing capacity for carbon monoxide (DLCO) was 51% predicted. Three patients died in follow-up, and two received lung transplantation. Restricted mean survival time for the entire cohort was 76 months.

According to the United States Bureau of Labor Statistics, the prevalence of firefighters employed in the Chicago-Naperville-Elgin metropolitan area in 2018 was 2.82 per 1,000 employed individuals (13,090 firefighters among 4,641,830 individuals). In our ILD cohort, the prevalence of firefighters was 11 per 1,000 (19 firefighters among 1,693 individuals); thus a prevalence rate ratio of 3.98 (95% CI: 2.40–6.22, *p* < 0.0001) was observed in firefighters in the ILD registry compared to firefighters within the working population of the Chicago area.

## Discussion

To our knowledge, this study is the first to describe ILD of various subtypes in firefighters not associated with the WTC attacks. Our analysis of a tertiary care center ILD cohort found a high rate of firefighters with a prevalence rate ratio approximately four times that of the general working population. These firefighters had a wide variety of ILD diagnoses, including CTD-ILD, IPF and CPFE. Other inhalational co-exposures were also common, suggesting that multiple exposures could have a synergetic effect on the development of ILD and highlighting the interconnected nature of these exposures in the real-world clinical setting.

This study found a disproportionately high rate of firefighters in our ILD patient population compared to the working population of the local metropolitan area. As these patients had all worked as firefighters for years prior to their ILD diagnosis, this finding suggests a potential causal mechanism between firefighting and ILD. Pulmonary fibrosis in WTC first responders has been described, with an increasing incidence related to increasing levels of exposure following the attacks (6). Systematically derived occupational exposure histories are needed in this patient population in order to assess for both a dose-response relationship as well as high-risk activities leading to ILD in firefighters.

We also found a wide variety of ILD subtypes in our firefighting cohort. Li and colleagues' description of pulmonary fibrosis in WTC first responders utilized patient self-report as a case definition, thus no determination of ILD subtype was able to be made (6). Sarcoidosis has been described in both WTC and non-WTC firefighters; however, this subgroup represented a small minority of ILD patients in our firefighting cohort (5, 10). This clinical diversity emphasizes the importance of asking about potentially remediable occupational inhalational exposures, such as firefighting, in all ILD patients regardless of clinical history or multidisciplinary diagnosis.

Inhalational co-exposures were common in firefighters with ILD. Many of these exposures overlapped in the same patients. While frequent co-exposures make assessment of any particular causal exposure especially challenging, they also emphasize the importance of a thorough, systematic exposure assessment on all ILD patients in order to elicit potential opportunities for remediation. Additionally, further work could assess whether multiple exposures increase the risk of severe ILD or other poor clinical outcomes in an additive or multiplicative fashion.

A substantial proportion of patients carried formal diagnoses of CTD in relation to their ILD or had positive serologic testing. In prior investigations of specific CTDs, the relation between tobacco and rheumatoid arthritis (RA) is the most consistent association described (11). The correlation between occupational exposure to mine dust, in particular silica, and autoimmune disorders, particularly systemic sclerosis, is well-described (12). More recently, a high prevalence of systemic autoimmune disease has been described in WTC first responders (7). Our understanding of the role of inhalational exposures in causing autoimmunity and ILD is limited, and precisely which substances are highest risk for causing disease in firefighters remains an area of active investigation.

This study has several limitations. First, the small number of patients and retrospective design make causal inferences difficult to elicit. We were unable to assess for specific job descriptions and exposure intensity for every patient. As this was an analysis of an already existing ILD cohort, we did not have a control population of patients without ILD or healthy firefighters with which to compare clinical features or risk factors. Nevertheless, our comparison with publicly available occupational data suggests the proportion of firefighters in our clinic was higher than that of the working population, and similar methods have been used in the past to suggest a potential causal mechanism between occupational exposures and lung disease (13). Although comparison of firefighting prevalence to the prevalence in the overall working population is imprecise given not every ILD patient seen in clinic is actively working, including the non-working population in the calculation of non-ILD firefighting prevalence would actually increase the prevalence rate ratio of firefighting seen in our ILD patients.

In summary, we found a higher proportion of firefighters in our ILD registry than was present in the working population, suggesting firefighting may be a novel risk factor for ILD. These patients had a wide variety of clinical presentations, ILD subtypes and autoimmune features. Further work should systematically assess for a dose-response relationship as well as high-risk occupational features in order to strengthen causal associations and identify potentially remediable exposure-related causes of ILD.

## Data Availability Statement

The data analyzed in this study is subject to the following licenses/restrictions:Protected Health Information. Requests to access these datasets should be directed to cathryn.lee@uchospitals.edu.

## Ethics Statement

The studies involving human participants were reviewed and approved by University of Chicago Institutional Review Board. The patients/participants provided their written informed consent to participate in this study.

## Author Contributions

All authors contributed to the study conception and design. Material preparation, data collection, and analysis were performed by CL, AA, and MS. The first draft of the manuscript was written by CL and all authors commented on previous versions of the manuscript. All authors read and approved the final manuscript.

## Conflict of Interest

CL receives grant funding from NIH (T32 HL007605). AA receives grant funding from NIH (K23 HL146942), American College of Chest Physicians, and the Pulmonary Fibrosis Foundation, honoraria for consulting, Speaker's Bureau, and advisory board from Boehringer Ingelheim. MS receives institutional support for research trials from Boehringer Ingelheim and Galapagos, honoraria for a post-graduate course from the American College of Chest Physicians, participates in an adjudication committee for Fibrogen, is the co-chair of the Industry Working Group and member of the planning committee for the Research Innovation Summit for the American Thoracic Society, and has received medical writing support from Boehringer Ingelheim. The remaining authors declare that the research was conducted in the absence of any commercial or financial relationships that could be construed as a potential conflict of interest.

## Publisher's Note

All claims expressed in this article are solely those of the authors and do not necessarily represent those of their affiliated organizations, or those of the publisher, the editors and the reviewers. Any product that may be evaluated in this article, or claim that may be made by its manufacturer, is not guaranteed or endorsed by the publisher.
